# Quercetin alleviates thiram induced tibial dyschondroplasia in broiler chicken through modulating oxidative stress and cecal microbiota

**DOI:** 10.3389/fvets.2026.1761862

**Published:** 2026-04-21

**Authors:** Rui Jiang, Yirui Yang, Ming Chi, Shuaiying Wang, Zhengyao Sheng, Jingyu Lyu, Tengteng Liu, Kaiwen Chen

**Affiliations:** 1 College of Veterinary Medicine, Nanjing Agricultural University, Nanjing, Jiangsu, China; 2 Veterinary Teaching Hospital, Nanjing Agricultural University, Nanjing, Jiangsu, China

**Keywords:** broiler, gut microbiota, oxidative stress, quercetin, tibial dyschondroplasia

## Abstract

**Introduction:**

Tibial dyschondroplasia (TD) is characterized by unmineralized cartilage plugs in the proximal tibial growth plate and is clinically associated with lameness and impaired growth performance in broilers. This study investigated the protective effects of quercetin, a natural flavonoid compound, against thiram-induced TD in broilers.

**Methods:**

A total of 180 one-day-old broilers were randomly assigned to a control group, a TD group, and a quercetin (QUE) group. TD was induced in the TD and QUE groups by feeding a diet containing 100 mg/kg thiram from days 4 to 7. The QUE group additionally received 600 mg/kg quercetin from the end of the adaptation period until the end of the trial. Growth performance, clinical signs, oxidative stress parameters, growth plate vascularization, cartilage-related gene expression, and gut microbiota composition were evaluated.

**Results:**

Compared with the control group, the TD group showed significant lameness, reduced growth performance, decreased serum ALP activity, T-AOC, and SOD levels, increased MDA levels, and reduced vascularization in the growth plate. qRT-PCR analysis demonstrated that the expression of antioxidant- and cartilage-related genes, including *Nrf2*, *HO-1*, *Col2a1*, and *ACAN*, was downregulated in the TD group, whereas quercetin supplementation upregulated the expression of these genes. Gut microbiota analysis further showed an increased relative abundance of Firmicutes and a decreased abundance of Proteobacteria in TD birds. Quercetin supplementation attenuated these negative changes, modulated the composition of the gut microbiota, and increased the abundance of beneficial bacteria, thereby improving intestinal health via the gut–bone axis and exerting a positive effect on the growth plate.

**Discussion:**

These findings indicate that quercetin alleviates thiram-induced TD in broilers, possibly by reducing oxidative stress, regulating cartilage-related gene expression, and improving gut microbiota homeostasis. Quercetin may therefore have potential as a feed additive for the prevention of TD, although the precise mechanisms require further investigation.

## 1 Introduction

Tibial dyschondroplasia TD is a prevalent metabolic skeletal disorder in fast growing boiler chickens, characterized by thickened, incompletely calcified, avascular white cartilage embolism in the proximal tibial growth plate. Up to 30% of broiler leg diseases were caused by TD (1). Affected birds show clinical signs like lameness and tibial deformities, substantially compromising both animal welfare and meat production (2). Current research recognizes that both genetic breeding and nutritional factors influence its incidence (3). For broilers of fast-growing genotypes, the rapid weight gain substantially elevates mechanical loading on leg joints, leading to the higher incidence of developing TD compared to slow-growing genotypes (4, 5). From a nutritional perspective, dietary calcium and phosphorus ratio constitute a primary factor influencing TD incidence rates, while other substances such as chloride and copper level also exert modulating effects (6–8). Microscopiclly, during normal growth plate development, chondrocytes undergo sequential differentiation from proliferative to hypertrophic stages, eventually forming mature tissue through endochondral ossification. Excessive chondrocytes apoptosis in the transition zone results in the failure of mature chondrocytes to complete terminal differentiation, leading to impaired mineralization and vascularization of the growth plate (1, 2, 9, 10). Oxidative stress is known to accelerate chondrocyte apoptosis and impair extracellular matrix development (11, 12). Consequently, scavenging oxygen free radicals has become a major research focus, alongside with inhibiting chondrocyte apoptosis, promoting angiogenesis and others. In this context, key biomarkers of oxidative stress and bone metabolism, including malondialdehyde (MDA), glutathione peroxidase (GSH-Px), superoxide dismutase (SOD), total antioxidant capacity (T-AOC), and alkaline phosphatase (ALP), are widely used to evaluate pathological status and therapeutic responses (13, 14). Furthermore, the expression of genes involved in antioxidant defense, such as Nrf2 and its downstream target HO-1, along with genes crucial for cartilage matrix integrity, such as type II collagen (Col2a1) and aggrecan (ACAN), serves as crucial molecular indicators for assessing chondrocyte function and differentiation capacity (15–17). *Nrf2* is a sensitive oxidative stress-responsive transcription factor involved in the transcriptional regulation of numerous antioxidant genes and plays a crucial role in cellular protection. HO-1 is an enzyme induced by *Nrf2*, and the products of its induced heme catabolism exhibit significant antioxidant and anti-inflammatory properties. The *Nrf2/HO-1* pathway plays a pivotal role in antioxidant defense against oxidative insults (18, 19). Col-II and ACAN are classic early chondrocyte markers and major components of the cartilage matrix, playing essential roles in the growth and development of the endochondral skeleton (2, 17). Previous studies have indicated that the loss of Col II and ACAN represents a classic histological feature of cartilage pathology, which has been consistently observed in osteoarthritic cartilage (20). Several Traditional Chinese Medicines (TCM)—such as Celastrol, Icariin, and Epigallocatechin Gallate—demonstrate efficacy in treating TD by mitigating oxidative stress (10). However, the broader application of these TCM is constrained by factors including high cost and absence of standardized evaluation criteria.

Quercetin, a naturally occurring flavonoid compound, primarily exists in glycosylated forms in various plants such as onions, apples, broccoli, and parsley (21). It exhibits diverse biological and pharmacological activities, including antioxidant, anti-inflammatory, antitumor, antiviral, and lipid-regulating properties (22). Recent studies have highlighted its regulatory effects on bone metabolism, positioning it as a therapeutic candidate for skeletal disorders like osteoporosis and osteoarthritis (23). Quercetin could also mitigate inflammation, chondrocyte apoptosis, and oxidative stress associated with bone diseases (22, 24). Limited research regarding quercetin’s application in broilers and its therapeutic efficacy against TD. We hypothesize that dietary quercetin addition can mitigate thiram-induced TD via modulating oxidative stress and gut microbiota. This study aims to evaluate quercetin’s therapeutic potential on growth performance, blood biomarkers, and cecal microbiota in broilers suffered from tibial dyschondroplasia.

## 2 Material and method

### 2.1 Ethics statement

This study was approved by the Nanjing Agricultural University’s ethics committee, Nanjing, People’s Republic of China (Approval No: NJAU. No20240711139).

### 2.2 Chicken management, diet and experimental design

**Figure 1:**
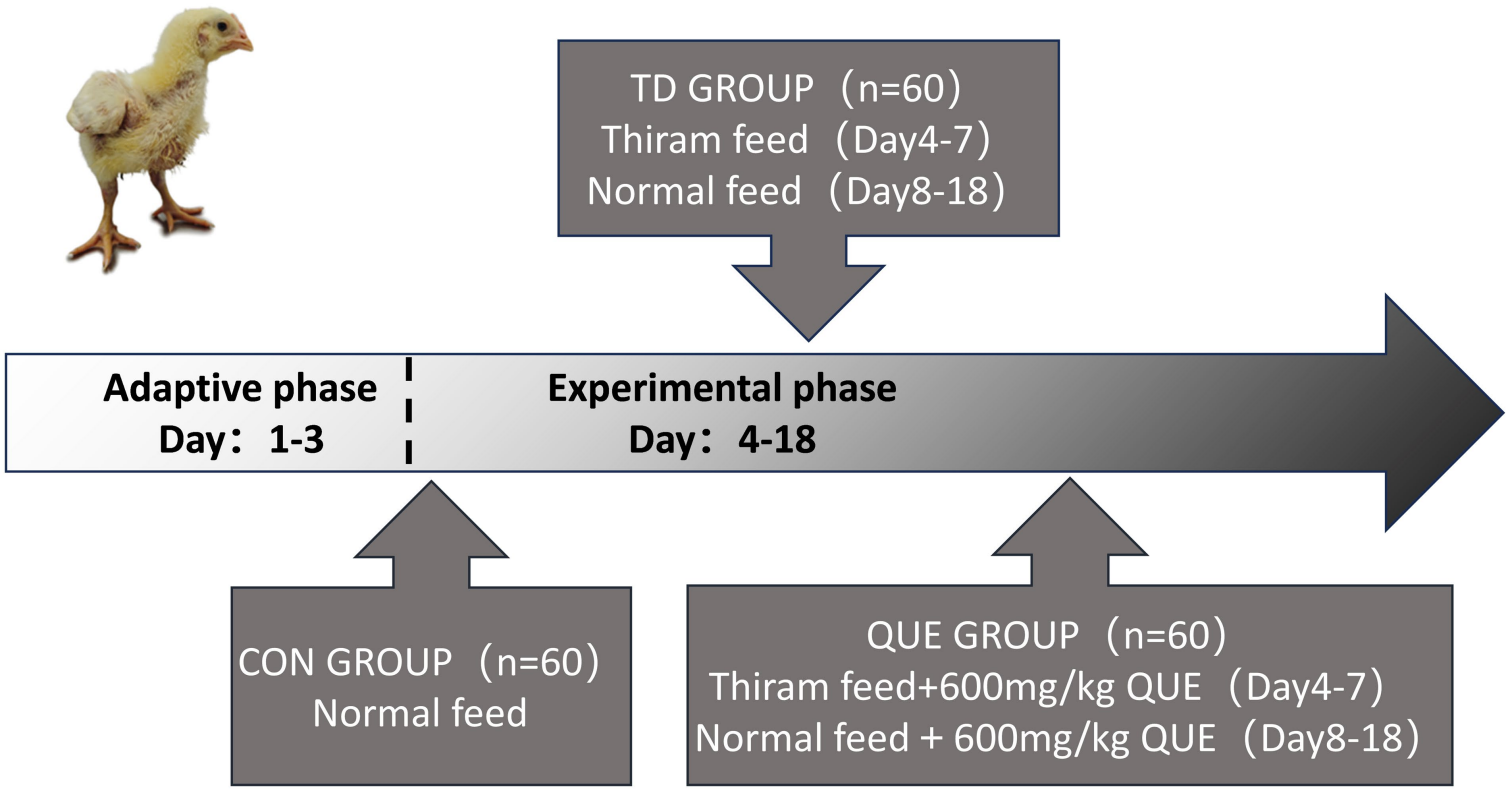
The experimental layout.

A total of 180 one-day-old male Arbor Acres broiler chickens (weighing 40 ± 5 g) were obtained from ShengLong Poultry Co., Ltd. (Suqian, China). The basal diet, formulated by Charoen Pokphand Group was provided throughout the experiment. After a three-day adaptation, all the broilers were randomly assigned to three groups (n = 60): Control (Con), Tibial Dyschondroplasia (TD) and Quercetin (Que). The overall experimental design is shown in Figure 1. During the entire 18 days of experiment, all groups received normal diet ad libitum while TD and QUE groups received additional 100 mg/kg of thiram (tetramethyl thiuram disulfide, purity: 97%, 22120A, Titan Co., Ltd., Shanghai, China) from days 4–7 to induce TD (25). QUE Quercetin (chemical formula: C_15_H_10_O_7_, purity: 97%, product code: Q817162-50 g, Macklin Biochemical Co., Ltd., Shanghai, China) was uniformly mixed with the diet for the QUE group using a feed mixer and offered in mash form (5 mm), achieving a final supplementation concentration of 600 mg per kilogram of feed (21, 26, 27). The environment temperature was maintained at 33–35 °C in the first week and steadily drops to 29 °C in the following weeks. Temperature and humidity were maintained within optimal ranges using digital hygrothermographs with continuous monitoring. Standardized illumination and ad libitum water access were maintained throughout the experiment.

Due to distinct intervention characteristics (e.g., quercetin coloration in feed), blinding of caregivers was not feasible. However, data analysis was conducted by blinded statisticians.

### 2.3 Sample collection and analysis of performance parameters

Growth performance parameters, including body weight, feed intake, and feed conversion ratio, were calculated for all groups. On days 7, 10, 14, and 18, twelve broilers per group were euthanized by cervical dislocation according to Zhang (25). Prior to euthanasia, blood samples (*n* = 6) were collected via jugular venipuncture. Whole blood was transferred into heparinized tubes and allowed to clot at ambient temperature for 30–60 min, followed by centrifugation at 3,500 × *g* for 15 min to isolate serum. Serum aliquots were stored at −20 °C until subsequent biochemical analyses. Tibial bones were also obtained from the broilers, and their weight, length, and width were measured using an electronic balance and a digital caliper (*n* = 5) to assess tibial development. The growth plate index (GPI) was calculated as follows: GPI = (growth plate height/tibial length) × 100%. This index can be used to assess the occurrence of tibial dyschondroplasia lesions(TDL). Five tibial bones from each group were fixed in 4% paraformaldehyde for Hematoxylin and Eosin (H&E) staining. The growth plate (*n* = 5) of tibial bones were split out and immediately snap-frozen in liquid nitrogen for subsequent RNA analysis. The remaining bones were stored at −80 °C for further study. On day 18, cecal contents were collected randomly from individuals from each group (*n* = 5) and stored at −80 °C for intestinal microbiota sequencing.

### 2.4 Serum biochemical and antioxidative activity analysis

Serum samples (*n* = 6) were analyzed using commercially available kits to assess biochemical indicators. Superoxide dismutase (SOD), malondialdehyde (MDA) and total antioxidant capacity(T-AOC)were determined to assess the oxidative stress status in the broilers. Additionly, levels alkaline phosphatase (ALP) were measured to evaluate skeletal development. Alkaline phosphatase (ALP) kits were sourced from Nanjing Jiancheng Bioengineering Institute (China); malondialdehyde (MDA) and superoxide dismutase (SOD) kits were obtained from Beyotime Biotechnology (China); total antioxidant capacity (T-AOC) kits were supplied by Beijing Solarbio Science & Technology Co., Ltd. (China). The detection limits for each indicator were as follows: SOD 0.5 U/mL, MDA 1 μM, T-AOC 0.5672 nmol/mL, and ALP 0 units/100 mL. Standard curves were established for all parameters according to the manufacturer’s kit instructions. All assays were performed according to the manufacturer’s instructions.

### 2.5 Gross and miscropic TD lesion observations

The fixed tibia collected on days 7, 10, 14, and 18 were randomly selected. Decalcification was performed using EDTA decalcification solution (Servicebio, G1105). Upon completion, the samples were dehydrated in gradient ethanol dilutions and cleared with xylene. Subsequently, paraffin embedding and sectioning were carried out. Hematoxylin–eosin staining was performed for 3–5 min, and finally examined and photographed under an optical microscope (Wuhan Service Biotechnology Co., Ltd.; Wuhan, China). The resulting longitudinal sections of the tibia were scanned using CaseViewer software for morphological analysis to assess vascularization patterns, cytomorphological features, and tissue organization as part of the histopathological evaluation.

### 2.6 RNA extraction and real-time quantitative polymerase chain reaction

Total mRNA was extracted from the tibial growth plate using TRIzol reagent (Accurate Biology Co., Ltd., Hunan, China)The mRNA quality was measured by a Nanodrop spectrophotometer, and cDNAs were subsequently synthesised by reverse transcription kit (Accurate Biology, China), and then qRT-PCR was performed on a Bio-Rad CFX96 Touch real-time system using SYBR Green qPCR Mix (Accurate Biology, China). The primer sequences (Supplementary Table S1)were designed on the official website of NCBI and synthesized by Shanghai Generay Biotech Co., Ltd.(Shanghai China).

### 2.7 16S rRNA sequencing

16S rRNA gene sequencing was conducted by Novogene Bioinformatics Technology Co., Ltd. to systematically analyze the composition and structure of the gut microbiota. This approach involved identifying microbial taxa, comparing community diversity and compositional differences, and assessing alterations in microbial composition across different experimental groups. Genomic DNA was isolated from samples using the CTAB/SDS method quantified through agarose gel electrophoresis, and diluted to a final concentration of 1 ng/μL using sterile water. Amplicons were generated using specific barcoded primers targeting the 16S V3-V4 regions. A 30 μL PCR reaction system was prepared using 15 μL Phusion High-Fidelity PCR Master Mix (New England Biolabs), 0.2 μM primers, and approximately 10 ng DNA template. PCR amplicons were combined at equimolar ratios, gel-purified (GeneJET Kit, Thermo Scientific), and constructed into Illumina libraries with the NEBNext Ultra™ Kit (NEB). Final sequencing was performed on HiSeq with 250-bp paired-end chemistry.

### 2.8 Statistical analysis

Data were analyzed using GraphPad Prism 10.0 software. Shapiro–Wilk test were conducted to check data normality. One-way and two-way ANOVA were used to determine significant differences. A *p*-value of ≤ 0.05 was considered statistically significant. Results are presented as mean ± standard deviation. Significant differences among the control, TD, and QUE groups are denoted by “*” (*p < 0.05, **p < 0.01, ***p < 0.001, ****p < 0.0001). Broiler gut microbiota analysis was performed using Novogene Cloud Platform’s online services^1^.

## 3 Result

### 3.1 Quercetin ameliorates the clinical symptoms of the TD-affected broilers

**Figure 2:**
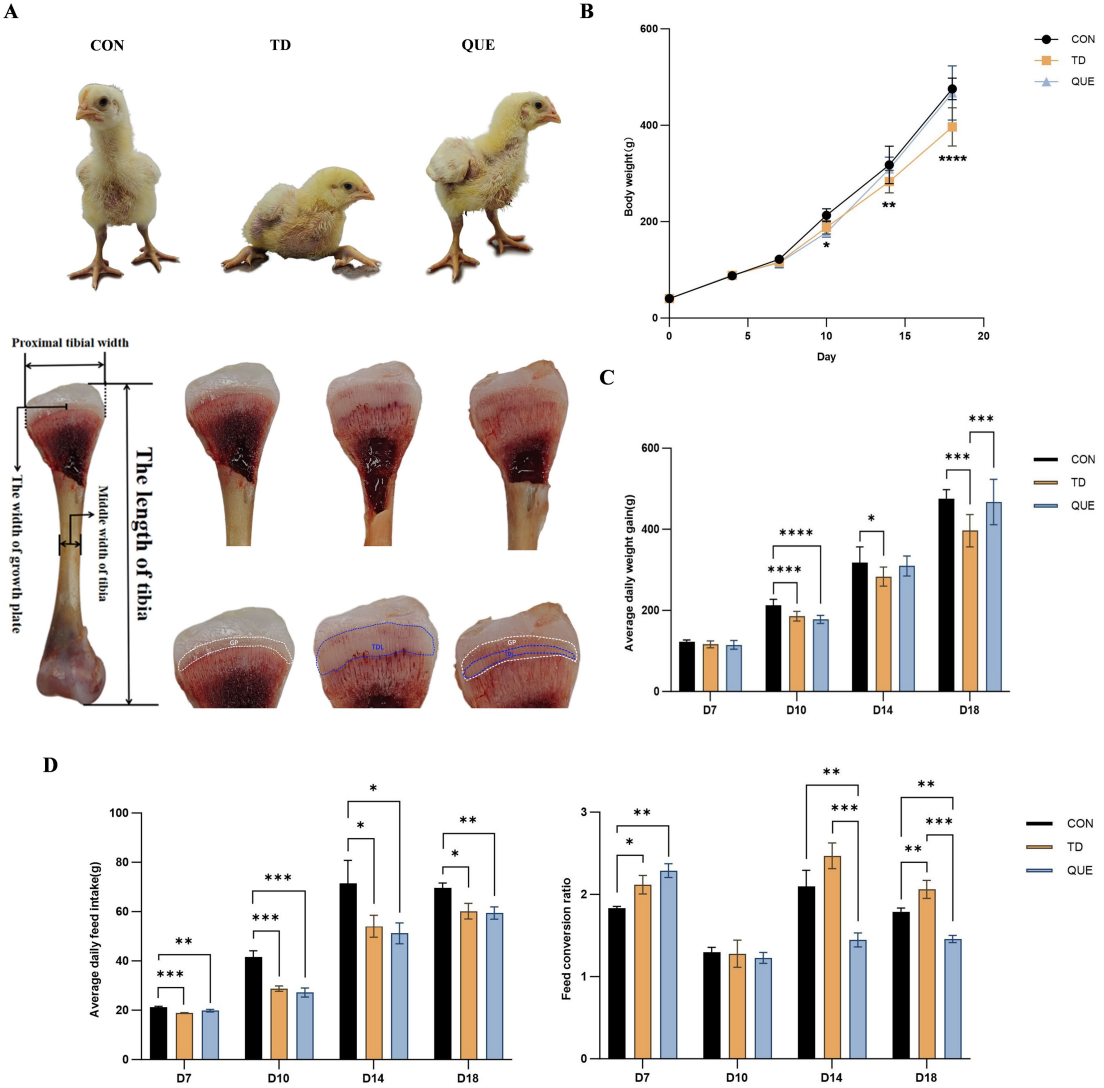
Tibial clinical presentation, morphological observation, and analysis of production performance. **(A)** Observation of clinical symptoms in 18 days. **(B)** Body weight. **(C)** Average daily weight gain (ADWG). **(D)** Average daily feed intake (ADFI) and feed conversion ratio (FCR). (*p < 0.05, **p < 0.01, ***p < 0.001, ****p < 0.0001).

Clinical symptoms were closely monitored through the experiment. Broilers in the TD group exhibited signs of straddle-like gait, difficulty walking, swollen joint, and fractures in severe cases. In contrast, the quercetin treatment group displayed less severe symptoms initially and showed faster recovery over time compared to the TD group (Figure 2A). Growth performance recorded during the experiment indicated that the body weight of the TD group gradually recovered to a level close to that of the control group (Figure 2B). The TD group showed significantly lower average daily weight gain (ADWG) and significantly higher feed conversion ratio (FCR) than the Control and QUE groups (*p* < 0.05) (Figures 2C,D). These findings demonstrate that quercetin alleviated the observable lameness, mitigated the decline in growth performance, and reduced growth plate lesions in broilers with TD.

### 3.2 Effect of quercetin on the tibial parameters

**Figure 3:**
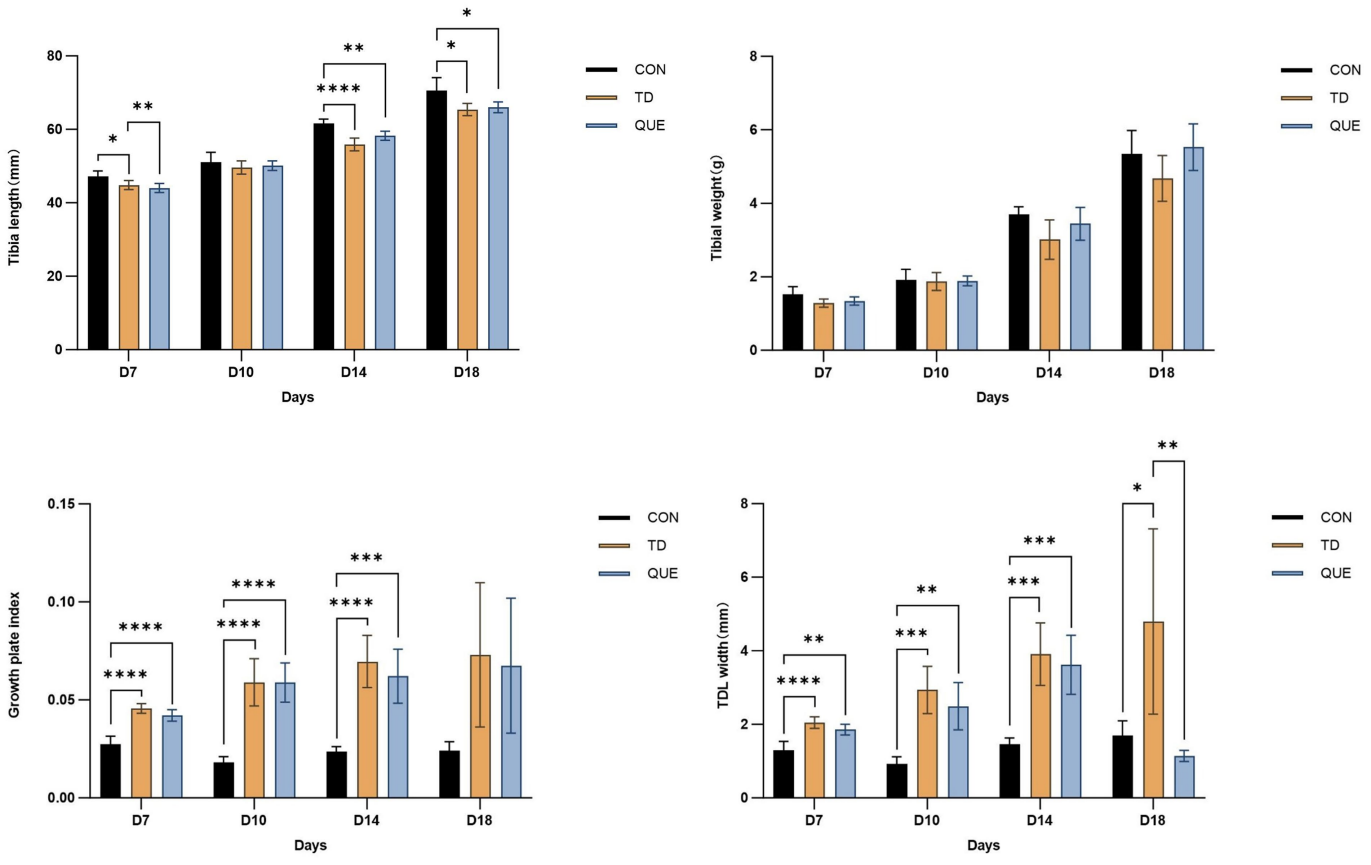
Tibial parameters (tibia length, weight, growth plate index, and TDL width) (*p < 0.05, **p < 0.01 ***p < 0.001).

Statistical analysis of tibial parameters showed that tibial length was significantly decreased in the TD group on days 14 and 18 (*p* < 0.05), whereas tibial weight showed a decreasing trend without significant difference, while the growth plate index was increased. While thiram induced a significant increase in TDL (*p < 0.05), this effect was effectively counteracted by quercetin treatment (Figure 3).

### 3.3 Histopathological examination of the tibial growth plate

**Figure 4:**
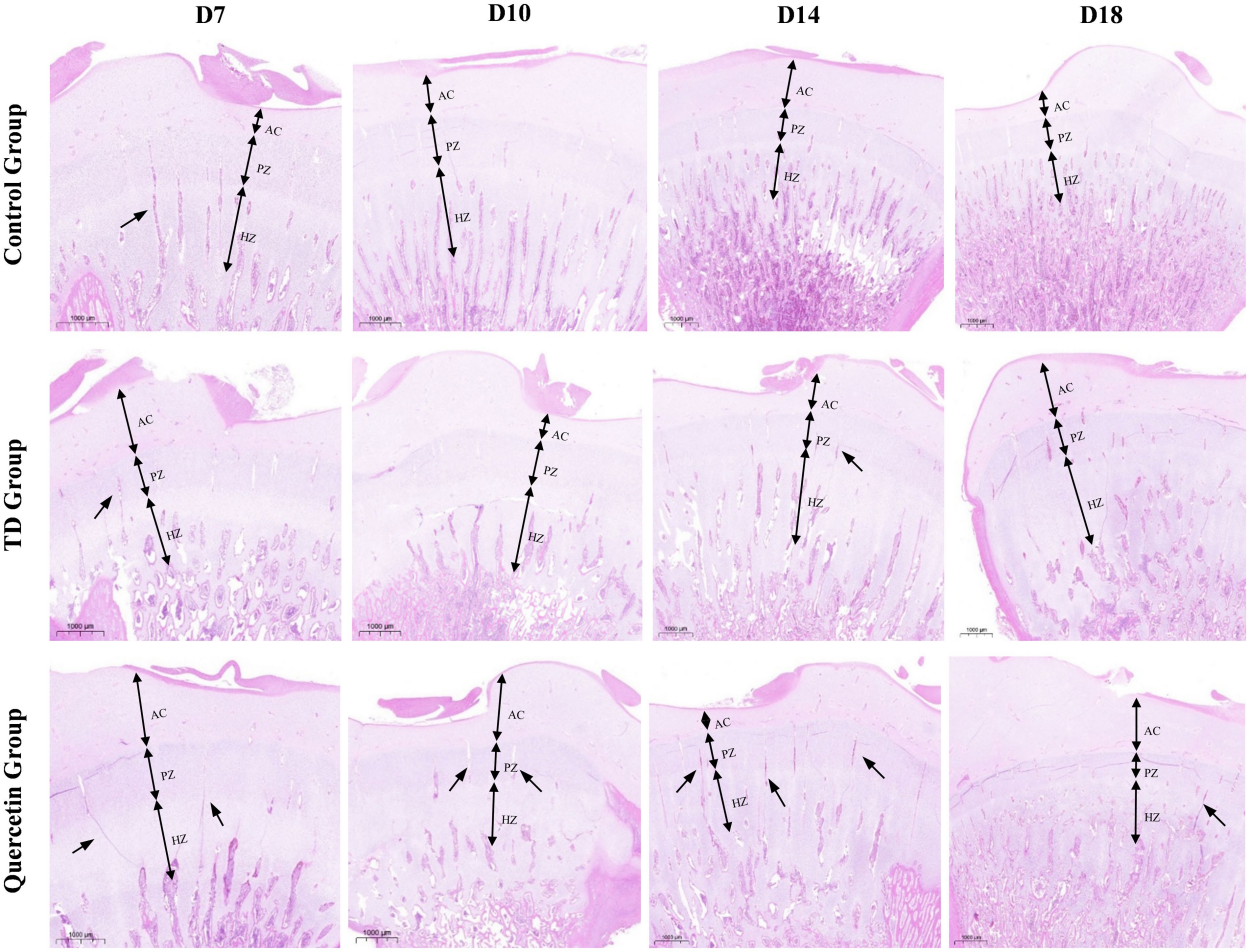
Hematoxylin and eosin (H&E) stained tibial growth plate. The black arrows depict blood vessels. Manifestation of enhanced blood vascularization in quercetin group. (AC, articular cartilage; PZ, proliferative zone; HZ, hypertrophic zone).

**Figure 5:**
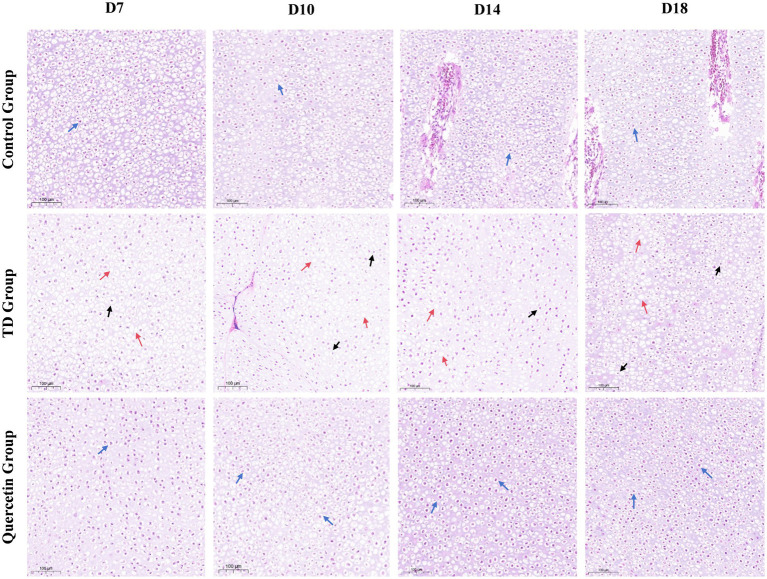
Histopathological examination and comparison of chondrocytes morphology in the hypertrophic zone of tibial growth plate (GP) among control, TD, and quercetin groups. The blue arrows depict regularly and tightly arranged chondrocytes with intact nuclei in control and quercetin groups. The red arrow points to the vacuolated chondrocytes and the black arrow points to the chondrocyte with a pyknotic nucleus in TD groups.

Histological analysis revealed distinct morphological differences among groups. In the control group, dense vascular distribution was observed in both the proliferative and hypertrophic zones of the growth plate. In contrast, the TD model group exhibited reduced vascular formation, with impaired vascular invasion into the growth plate. Remarkably, the QUE group showed significant improvement in angiogenesis, with abundant vascular networks detectable in the growth plate from days 10 to 18 (Figure 4). Cellular morphology further highlighted these disparities: chondrocytes in the control group displayed regular alignment, whereas the TD group presented widespread cellular abnormalities, including vacuolation, altered morphology, and nuclear pyknosis. In contrast, the QUE group exhibited improved cellular architecture, characterized by a more regular cell arrangement and a reduced number of dead cells relative to the TD group (Figure 5).

### 3.4 Effect of quercetin on serum biochemical and antioxidant parameters

**Figure 6:**
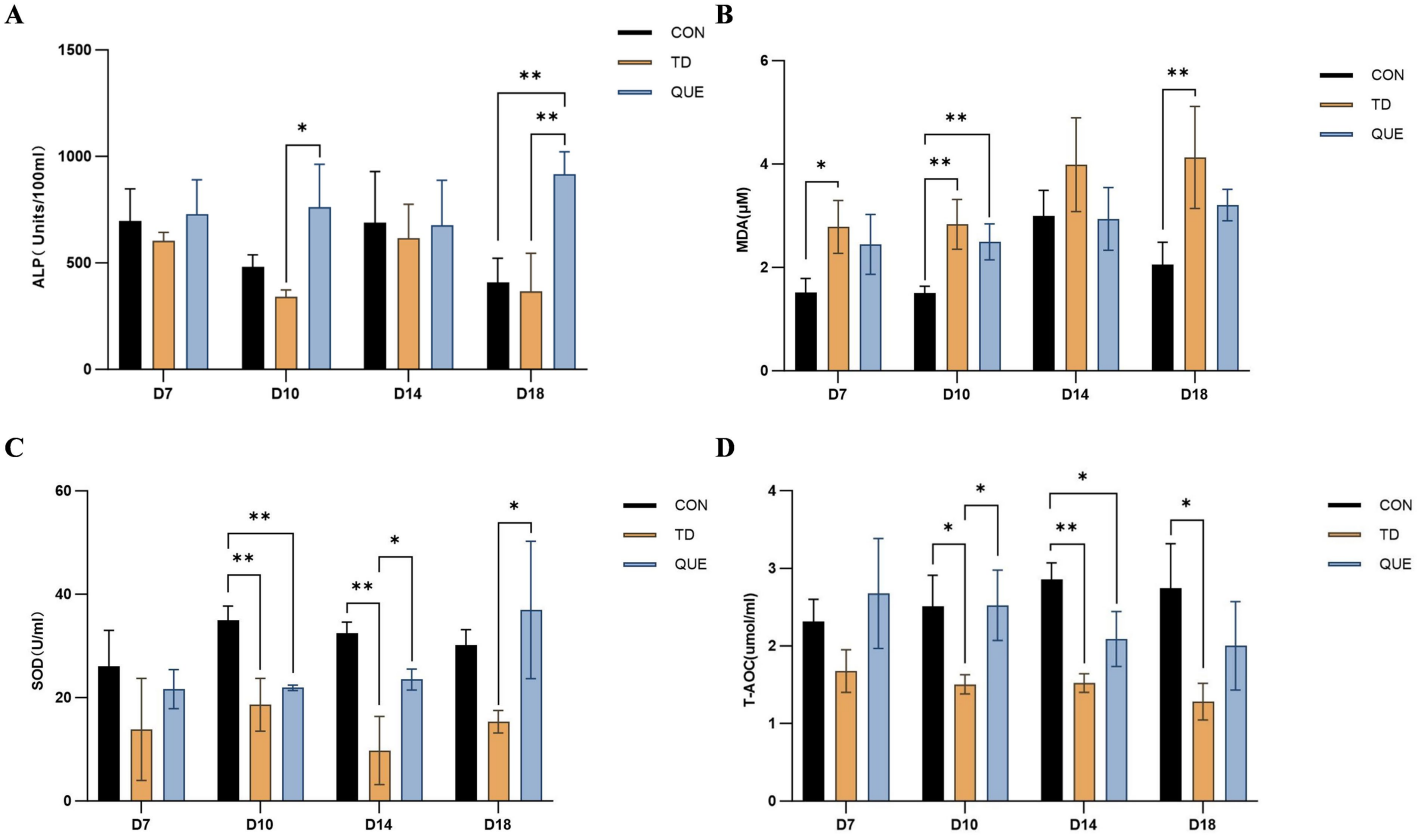
Analysis of serum biochemical and antioxidant parameters among control, TD, and quercetin groups on specific days of sampling. **(A–D)** ALP, MDA, SOD, and T-AOC. Quercetin treatment restored these parameters close to control level. (*p < 0.05, **p < 0.01).

Serum biomarker and biochemistry profiles worsened after the administration of thiram from days 4 to 7. Biochemical assays showed that TD birds exhibited a marked decline in ALP activity. This decline was counteracted by quercetin treatment, which significantly elevated ALP activity (*p* < 0.05) to levels exceeding the control group by day 18(Figure 6A). Concurrently, malondialdehyde (MDA) levels showed persistent elevation throughout the experiment. Administration of quercetin attenuated the thiram-induced increase in MDA levels (Figure 6B). Furthermore, the activities of SOD and T-AOC, which were suppressed in the TD group, returned toward levels comparable to those of the CON group following quercetin treatment (Figures 6C,D).

### 3.5 Regulation of Nrf2/HO-1 and cartilage matrix gene (Col2a1/ACAN) expression by quercetin in TD broilers

**Figure 7:**
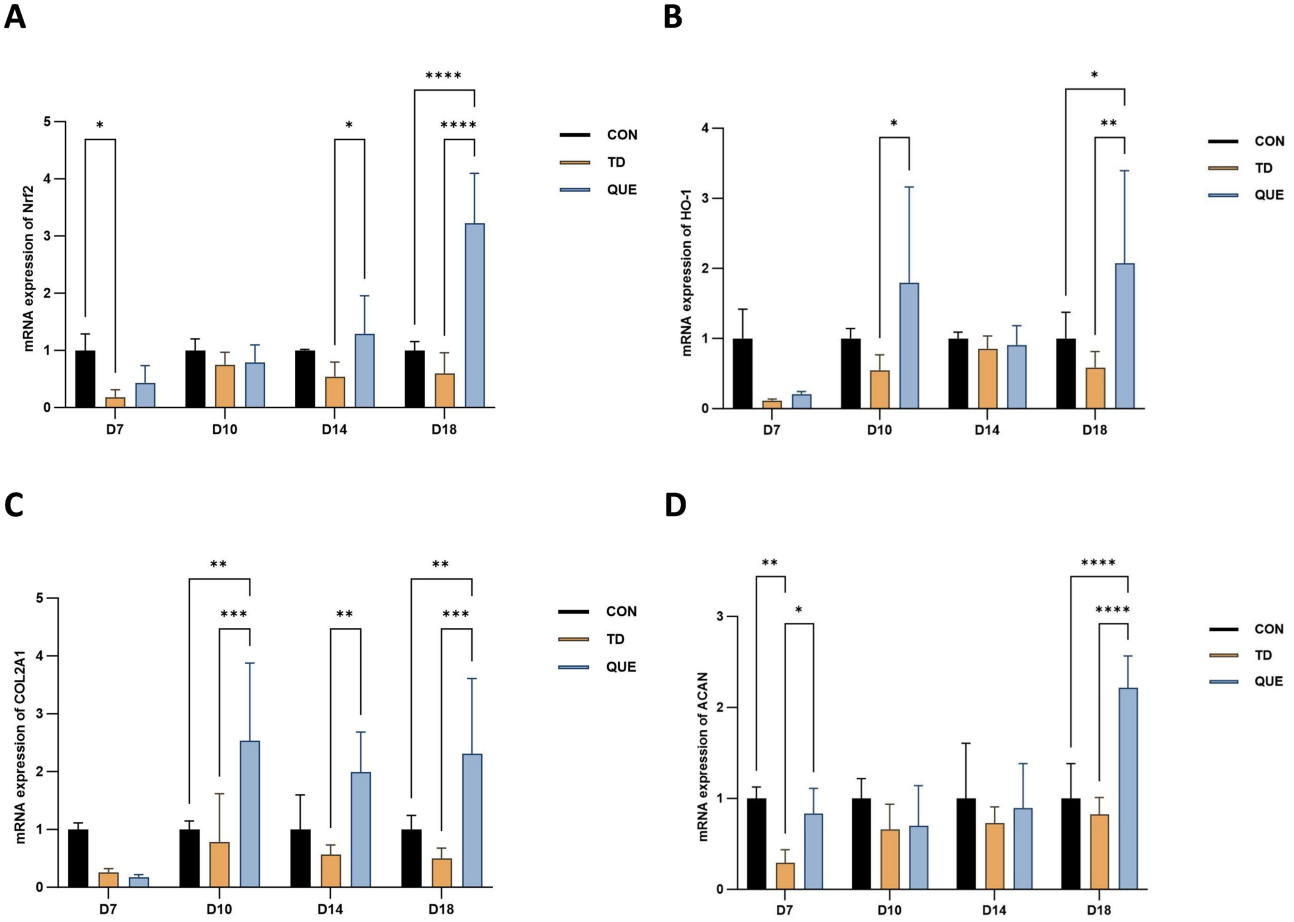
The mRNA expression of *Nrf2*, *HO-1*, *Col2a1* and ACAN was assessed by RT-qPCR. **(A)** Illustrates the mRNA expression of *Nrf2*. **(B)** Illustrates the mRNA expression of *HO-1*. **(C)** Illustrates the mRNA expression of *Col2a1*. **(D)** Illustrates the mRNA expression of *ACAN*. (*p < 0.05, **p < 0.01,. ***p < 0.001, ****p < 0.0001).

To investigate the expression changes of relevant genes in TD broilers under quercetin intervention, the mRNA levels in each group were measured on days 7, 10, 14, and 18. The results showed that in the TD group, the expression of *Nrf2* and *HO-1* was suppressed, with the downregulation of *Nrf2* being statistically significant on day 7 (*p* < 0.05). In contrast, continuous quercetin intervention promoted the expression of these genes in the QUE group, and by day 18, the expression levels of *Nrf2* and *HO-1* had significantly surpassed those in the CON group (*p* < 0.01). Furthermore, the expression of the cartilage matrix genes *Col2a1*(*p* < 0.05) and *ACAN* (*p* < 0.01)was also consistently downregulated in the TD group, with *ACAN* showing a significant decrease as early as day 7,. Quercetin intervention significantly upregulated the expression of both genes in the QUE group, and this upregulation exhibited a time-dependent enhancement. Specifically, the expression of *Col2a1* (*p* < 0.05) was significantly elevated on days 10, 14, and 18, while that of *ACAN* was significantly elevated on day 7 and day 18 (*p* < 0.01). By the later stages of the experiment, the expression levels in the QUE group had become significantly higher than those in the CON group (*p* < 0.01) (Figures 7A–D).

### 3.6 Cecal microbiota

**Figure 8:**
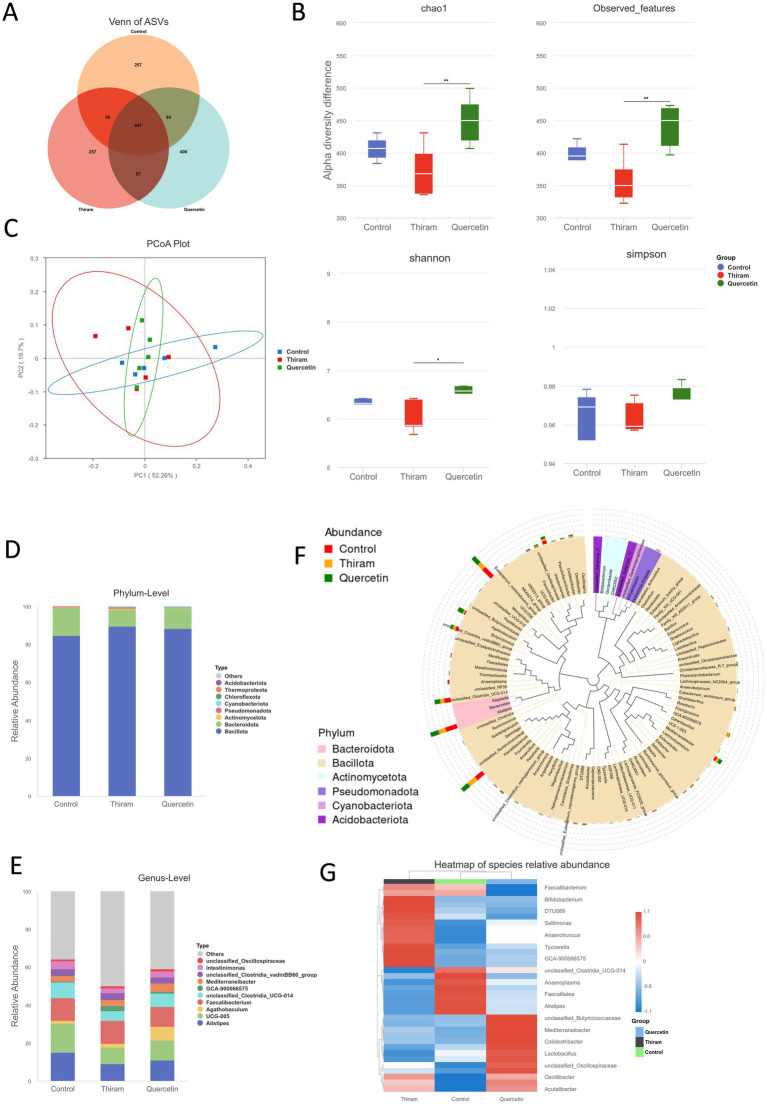
Diversity analysis among different groups and microbial abundance analysis. **(A)** Venn based on ASVs level. **(B)** Alpha diversity analysis based on Chao1, observed features, Shannon, and Simpson index. **(C)** PCoA analysis (*n* = 5/group). **(D)** Microbial composition at the phylum level. **(E)** Microbial composition at the genus level. **(F)** Genus-level phylogenetic tree. **(G)** Genus-level species abundance heatmap.

Recent investigations have uncovered a mechanistic link between the intestinal microbiota and skeletal disorders in poultry (28). Published studies have defined a gut–pancreas–bone axis that modulates systemic glucose homeostasis in TD and have mapped microbiota-driven shifts in short-chain fatty acid metabolomes, collectively indicating that the gut microbiome exerts a crucial role in TD pathogenesis (29, 30). To evaluate the effects of quercetin on cecal microbiota, 16S rRNA gene sequencing was performed to characterize microbial composition across groups. The Venn diagram revealed distinct microbial community patterns among the three groups (Figure 8A). A total of 447 amplicon sequence variants (ASVs) were shared across all groups. Group-specific ASVs were identified as follows: 257 in the control group, 257 in the TD group, and 400 in the quercetin-treated group, indicating a higher microbial uniqueness in the quercetin group.

#### 3.6.1 Diversity analysis of intestinal microbiota

To assess gut microbial diversity across treatments, both alpha- and beta-diversity analyses were performed. Alpha-diversity metrics, including Chao1, observed features, Shannon, and Simpson indices, indicated significantly lower microbial richness and evenness in the TD group compared to the control. Quercetin supplementation not only restored but also significantly enhanced microbial diversity (*p* < 0.01, Figure 8B). Beta-diversity analysis, based on principal coordinate analysis (PCoA), revealed no distinct clustering among groups, suggesting that TD primarily reduced within-sample diversity rather than altering overall community structure (Figure 8C).

#### 3.6.2 Analysis of intestinal microbial composition

To investigate the effects of quercetin on gut microbiota composition in broilers, a comprehensive taxonomic analysis of microbial community structures was conducted. At the phylum level, *Bacilliota* and *Bacteroidota* were identified as the dominant phyla across all experimental groups (Figure 8D). Compared with the control group, the TD group exhibited a significant increase in the relative abundance of *Bacilliota*, *Actinomycetota*, *Pseudomonadota*, and *Cyanobacteriota*, accompanied by a notable decrease in *Bacteroidota*.

At the genus level, the top 10 most abundant genera included *Alistipes*, *UCG-005*, *UCG-014*, *Faecalibacterium*, and *Intestinimonas,* which collectively constituted a substantial proportion of the microbial community. The TD group showed a marked enrichment of *Faecalibacterium*, while the abundances of *Alistipes*, *UCG-005*, *UCG-014*, and *Intestinimonas* were significantly reduced. Quercetin intervention effectively counteracted these shifts (Figures 8E,G). The genus-level phylogenetic tree further supported these findings, illustrating the taxonomic relationships and shifts among dominant genera (Figure 8F).

#### 3.6.3 Differential analysis and KEGG pathway prediction

**Figure 9:**
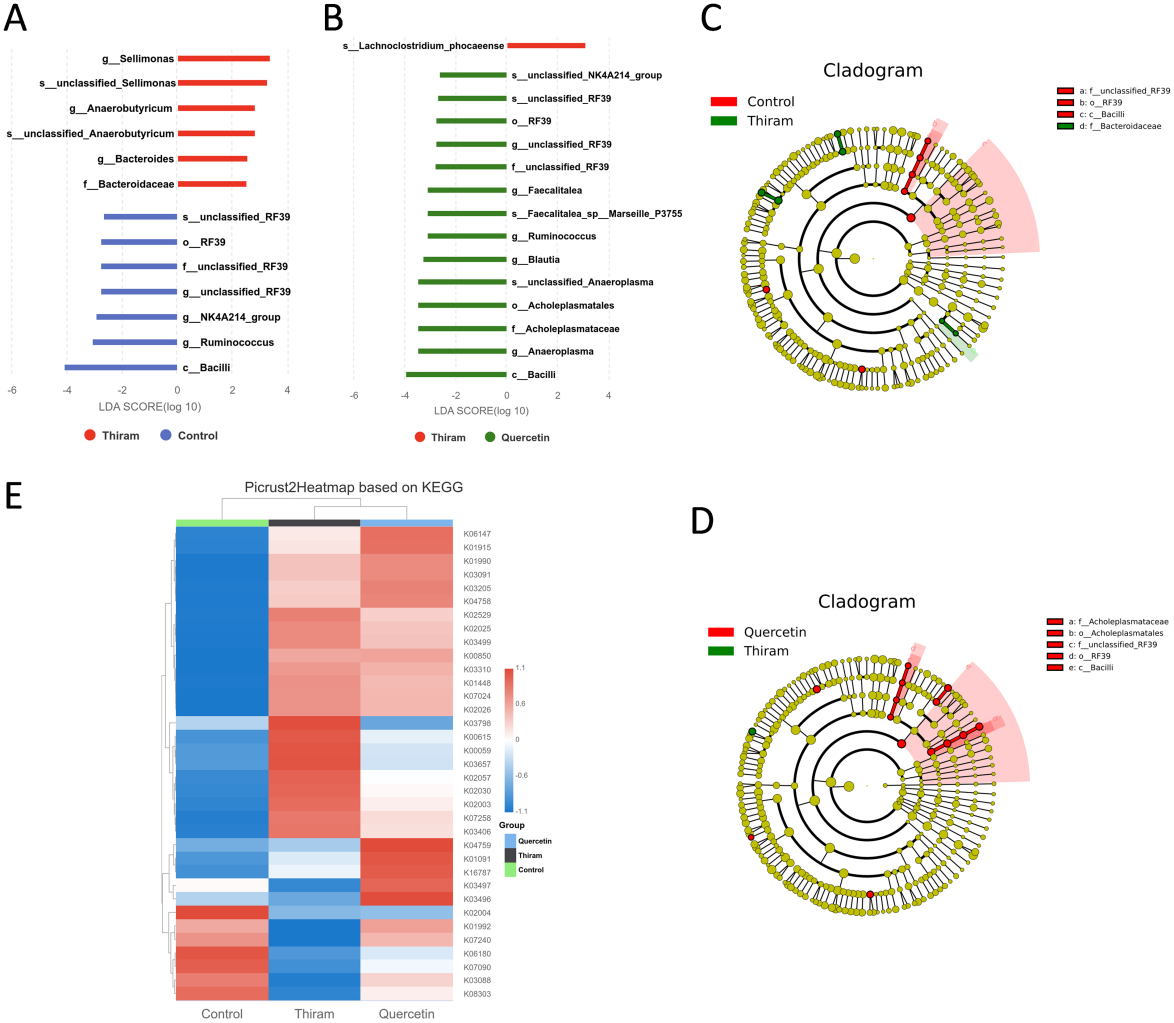
LEfSe analysis and functional prediction. **(A)** LEfSe analysis between control and Thiram group. **(B)** LEfSe analysis between Thiram and quercetin group. **(C)** Histogram of LDA value distribution between control and Thiram group. **(D)** Histogram of LDA value distribution between Thiram and quercetin group. **(E)** PICRUSt2-based functional prediction against the KEGG pathway database.

Linear discriminant analysis effect size (LEfSe) was employed to identify statistically significant microbial biomarkers across taxonomic levels (LDA score > 2, *p* < 0.05). The results revealed significant enrichment of specific taxa between pairwise group comparisons (Figures 9A–D). Compared with the control group, the TD group exhibited significant enrichment of *Sellimonas*, *Anaerobutyricum*, and *Bacteroides*, whereas the control group showed higher abundance of RF39, *Ruminococcus*, and *Bacilli*. In contrast, compared with the TD group, the quercetin group displayed significant enrichment of *NK4A214*, RF39, *Faecalitalea*, *Ruminococcus*, *Blautia*, and *Bacilli*, many of which overlapped with the taxa enriched in the control group. These findings suggest that quercetin partially restores beneficial microbiota, thereby exerting a therapeutic effect.

To explore the potential mechanisms underlying quercetin-mediated modulation of gut microbiota, functional prediction was performed using PICRUSt2 against the KEGG pathway database. KEGG pathway analysis revealed a marked up-regulation of sugar transport and carbohydrate metabolism modules (K02529, K02025, K07024, K02026, K02057) in the TD group (Figure 9E). This up-regulation was attenuated following quercetin treatment. The observed modulation is consistent with the recent view that blood glucose homeostasis is a factor in TD (30). In addition, metal-ion transport pathways (K02030, K03406, K07240) were also altered in TD, implying a link between TD and metal-ion-induced oxidative stress, which may be modulated by the antioxidant function of quercetin.

## 4 Discussion

Tibial dyschondroplasia (TD), a major metabolic cartilage disease affecting the tibia bone in avian species, which severely impacts broiler carcass quality and causes significant economic losses in the poultry industry (31). Our study focuses on the therapeutic efficacy of quercetin against thiram-induced TD. Thiram has been well-established as an effective experimental agent for inducing tibial dyschondroplasia (TD) in broilers. Extensive literature indicates that the thiram-induced TD model closely resembles naturally occurring TD in both physiological and pathological characteristics, thus serving as a valuable tool for studying the pathogenesis of the disease (8, 32). The experimental design encompassed adaptation, modeling, acute onset, and recovery phases, allowing for systematic observation of the disease progression of tibial dyschondroplasia (TD) in broilers, with the aim of comprehensively elucidating its initiation and developmental patterns. Previous research has highlighted quercetin—a natural flavonoid with antioxidant, anti-inflammatory, and anti-aging properties—as a promising component in bone-related disorders (24). Studies demonstrate that dietary addition with 400 mg/kg quercetin improves egg production, feed efficiency, liver function, follicular development, and reproductive organ performance in laying hens (33, 34), while 600 mg/kg quercetin enhances carcass quality, growth performance, immune function, and antioxidant capacity in broilers (21, 26). Therefore, 600 mg/kg quercetin was referred as the treatment dose to evaluate its therapeutic effects on TD in broilers. Results revealed that quercetin alleviated adverse symptoms such as lameness, splayed legs, skeletal deformities, reduced feed intake, and weight loss in TD-affected birds. HE staining results revealed disorganized cell arrangement in the TD group, with increased incidences of vacuolation and nuclear pyknosis. Concurrently, reduced angiogenesis was observed in the TD group. In contrast, the quercetin group was associated with restoration of cellular morphology, reduction in vacuolated and pyknotic cells, and increase in vascularization. These findings indicate that quercetin effectively alleviates TD-induced cytopathological alterations and enhances vascularization capacity within the growth plate.

Prolonged thiram exposure (e.g., 100 mg/kg in feed) suppresses antioxidant activity, increases reactive oxygen species (ROS), induces gut microbiota dysbiosis and hepatic lipid metabolism disorders (35, 36). Our study confirmed that thiram-induced TD significantly elevated MDA levels, indicating systemic oxidative stress. Quercetin counteracted these effects by reducing MDA, restoring SOD activity and enhancing the total antioxidant capacity demonstrating its potent antioxidant capacity (37–39). This experiment also measured the mRNA expression levels of two oxidative stress-related genes, *Nrf2* and *HO-1*. Analysis of the results revealed that the mRNA expression levels of *Nrf2* and *HO-1* were consistently downregulated across all four time points in the TD group, indicating that thiram exacerbates oxidative stress in broilers with TD by suppressing this pathway. In contrast, quercetin treatment effectively attenuated the reduction in mRNA expression of both genes, with expression levels surpassing those in the CON group by day 18. Together with hepatic antioxidant indices, these findings suggest that quercetin alleviates thiram-induced oxidative stress and ameliorates TD symptoms, likely through activation of the *Nrf2/HO-1* pathway.

Alkaline phosphatase (ALP), a marker of osteoblast activity critical for bone mineralization, is reduced in bone-related disorders such as osteoporosis (40). While ALP levels exhibited a non-significant decrease in the TD group, quercetin treatment resulted in a significant elevation of serum ALP by day 18, surpassing control levels. This change coincided with a recovery of oxidative balance, which could be linked to the improvements in clinical signs and performance metrics. This study also revealed that the mRNA expression levels of Col2a1 and ACAN in the growth plate of TD broilers were downregulated, indicating that TD impairs normal chondrocyte function and extracellular matrix structure. Dietary supplementation with quercetin significantly upregulated the mRNA expression of both genes. These results suggest that quercetin may alleviate TD symptoms by regulating the expression of chondrogenic differentiation and extracellular matrix genes, thereby promoting cartilage regeneration and repair.

Recently, the role of the gut microbiota in neutrient metabolism, immune regulation and bone health has been studied (41). Previous study have demonstrated the potential of quercetin in improving postmenopausal osteoporosis, which may effectively reduce bone loss in ovariectomized rats through the “gut microbiota-short chain fatty acid (SCFA)-inflammation” signaling pathway (42, 43). TD is influenced by numerous factors, and the gut microbiota may involve complex interactions between nutrition and hormone regulation of the growth plate (44).

Further analysis of the microbial composition of each group revealed that *Bacilliota and Bacteroidota* were the common dominant phyla of all three groups consist with previous studies (45, 46). Notably, dynamic changes in *Bacilliota* and *Bacteroidota* have been closely associated with disease development (47). Study have shown that during the progression of rheumatoid arthritis, the elevated species mainly come from the *Bacilliota* phylum, while the decreased species mainly come from the *Bacteroidota* phylum (48). In a recent OVX-rat study (22), quercetin reversed the elevation of *Bacilliota* and reduction of *Bacteroidota*, significantly lowering the F/B ratio which is commonly associated with intestinal health and metabolic disorders, with a lower ratio favoring increased synthesis and secretion of SCFAs by intestinal flora, and enriching SCFA-producing taxa (42). Consistent with earlier reports, our results demonstrated an increased relative abundance of *Bacilliota, Actinomycetota, Pseudomonadota* in the TD group (28, 30, 45, 49). Quercetin administration effectively reduced the abundance of *Bacilliota.* Compared with the control group, the proportion of *Bacteroidota* decreased in the TD group and increased in the QUE group. These phylum-level shifts were linked to increased intestinal SCFAs, enhanced intestinal barrier function, and improved bone microarchitecture, indicating that quercetin-driven *Bacilliota* decrease and Bacteroidota increase might contribute to its gut-bone axis protective effect.

At the genus level, the TD group increased the abundance of *Faecalibacterium* and decreased that of *Alistipes* and *Intestinimonas*. *Faecalibacterium* maintains intestinal epithelial energy homeostasis and exerts anti-inflammatory and barrier-protective effects via the butyrate–PPARγ axis (50). Alistipes and Intestinimonas contributes to host nutrition and energy supply through SCFAs production, while regulating immune cell activity and maintaining intestinal homeostasis (51). These results may represent a compensatory mechanism to preserve intestinal barrier function. LEfSe analysis revealed that the abundance of *Ruminococcus* was significantly higher in both the control and quercetin groups compared with the TD group. As key decomposers of cellulose and polysaccharide substrates, *Ruminococcus* serves as crucial SCFAs producers associated with intestinal mucus barrier function and anti-inflammatory effects (52–54). Our findings align with established evidence: decreased Ruminococcaceae levels associate with osteoporosis progression, implicating gut microbiota dysbiosis in bone density regulation (55). In addiiton, Gao et al. found that *Ganoderma lucidum* polysaccharides could significantly enhance the relative abundance of cecal *Ruminococcaceae* in broilers, thereby improving lipid metabolism, antioxidant capacity, and intestinal microbiota composition (56). Similarly, our experiment revealed that quercetin could increase the relative abundance of *Ruminococcaceae* in the cecal microbiota of TD broilers.

PICRUSt2 revealed that TD altered pathways involved in carbohydrate transport and metabolism as well as metal-ion transport. Quercetin reversed these changes, indicating that it may lower blood glucose and alleviate oxidative stress in broilers through the gut-pancreas axis and gut-bone axis, thereby exerting a therapeutic effect (28, 30, 39, 49).

Based on the results mentioned above, quercetin demonstrates therapeutic potential for TD management, but the molecular mechanisms underlying its microbiota-mediated regulation of bone metabolism remain incompletely elucidated. Future studies should employ multi-omics integrative analyses to explore the dynamic regulation of the host-microbiota interaction network. Fecal microbiota transplantation (FMT) technology can be combined to verify the role of specific microbiota functions in bone formation, so as to deeply explore the potential mechanism of quercetin in regulating the intestinal microbiota and improving bone metabolism in TD broilers.

There were some limitations in the present study. The effects of specific bacteria regulated by quercetin on the inflammatory responses of the gut were not investigated. Experiments *in vivo* investigating the role of some specific bacteria on gut inflammatory responses in broilers might help clarify the anti-inflammatory effects of dietary quercetin via microflora.

## 5 Conclusion

Quercetin emerges as an effective herbal intervention that ameliorates lameness antioxidative stress, while modulating gut microbiota in TD-affected broilers by improving growth parameters such as body weight, feed efficiency, and tibial metrics.

Our findings underscore quercetin’s therapeutic potential for thiram-induced TD, warranting further mechanistic studies to elucidate its action pathways and advance prevention or treatment strategies for broiler tibial dyschondroplasia. The therapeutic efficacy of quercetin in broilers with spontaneously occurring tibial dyschondroplasia warrants further verification.

## Data Availability

The datasets presented in this study can be found in online repositories. The name of the repository and the accession number are as follows: NCBI BioProject, PRJNA1374220.

## Footnotes

1
https://magic.novogene.com

## Supplementary material

SUPPLEMENTARY TABLE S1 Primer sequences used for RT-qPCR analysis.
