# Supplementary material for: Quercetin alleviates thiram induced tibial dyschondroplasia in broiler chicken through modulating oxidative stress and cecal microbiota
Source: Front Vet Sci. 2026 Apr 21;13:1761862. doi: 10.3389/fvets.2026.1761862 (PMC13139136; doi:10.3389/fvets.2026.1761862)
Supplement: SUPPLEMENTARY TABLE S1 — Primer sequences used for RT-qPCR analysis. [file Table_1.pdf]

Gene	Forward of primer sequence (5’→3’)	Reverse of primer sequence (5’→3’)
GAPDH	CACTGTCAAGGCTGAGAACG	TGATAACACGCTTAGCACCA
CoL2a1	ACCTACAGCGTCTTGGAGGA	ATATCCACGCCAAACTCCTG
ACAN	TGCAAGGCAAAGTCTTCTACG	GGCAGGGTTCAGGTAAACG
Nrf2	AGTGACCCAGTCTTCATTTC	TCTTCCCAAACTTGCTCTAT
HO-1	AAACTTCGCAGCCACACAAC	GACCAGCTTGAACTCGTGGA
